# Imaging Endocytosis Dynamics in Health and Disease

**DOI:** 10.3390/membranes12040393

**Published:** 2022-04-01

**Authors:** Erica Tagliatti, Katia Cortese

**Affiliations:** 1 Laboratory of Pharmacology and Brain Pathology, Humanitas Clinical and Research Center, Via Manzoni 56, 20089 Milano, Italy; 2 Department of Clinical and Experimental Epilepsy, UCL Queen Square Institute of Neurology, University College London, London WC1E 6BT, UK; 3 Cellular Electron Microscopy Laboratory, Department of Experimental Medicine (DIMES), Human Anatomy, Università di Genova, Via Antonio de Toni 14, 16132 Genova, Italy

**Keywords:** endocytosis, protein receptor, disease, fluorescence, electron microscopy, super-resolution microscopy, correlative microscopy

## Abstract

Endocytosis is a critical process for cell growth and viability. It mediates nutrient uptake, guarantees plasma membrane homeostasis, and generates intracellular signaling cascades. Moreover, it plays an important role in dead cell clearance and defense against external microbes. Finally, endocytosis is an important cellular route for the delivery of nanomedicines for therapeutic treatments. Thus, it is not surprising that both environmental and genetic perturbation of endocytosis have been associated with several human conditions such as cancer, neurological disorders, and virus infections, among others. Over the last decades, a lot of research has been focused on developing advanced imaging methods to monitor endocytosis events with high resolution in living cells and tissues. These include fluorescence imaging, electron microscopy, and correlative and super-resolution microscopy. In this review, we outline the major endocytic pathways and briefly discuss how defects in the molecular machinery of these pathways lead to disease. We then discuss the current imaging methodologies used to study endocytosis in different contexts, highlighting strengths and weaknesses.

## 1. Introduction

Endocytosis is a shared process by which molecules, proteins, lipids, and liquids are sorted inside the cell via formation of intermediate vesicles [1]. Vesicle formation occurs at the plasma membrane, where ligand receptors, binding proteins, and structural proteins are localized. After their internalization, the vesicles containing protein receptors or soluble molecules undergo a round of recycling, eventually leading to the fusion of the vesicle with an intracellular organelle. Such a process is an essential hallmark in all cell types—it regulates major cellular functions such as antigen presentation, intracellular signaling cascades, cell polarity, and synaptic transmission. Moreover, it is required to remove aged and dead cells from the body and is part of the defense against microbes. Given its importance, it is not surprising that even subtle perturbations affecting the endocytic machinery often impair cell function and cause several pathological conditions, such as cancer, and neurological and storage diseases [2,3,4,5]. Finally, endocytosis represents an important cellular route for targeted drug-delivery in many diseases [6]. The term “endocytosis” was used for the first time by Christian De Duve in 1963, but the emerging concept of internalization/endocytosis goes way back in time—to the end of the nineteenth century. The 1908 Nobel Prize zoologist Elie Metchnikoff was the first to identify phagocytes and recognized the importance of phagocytosis as part of the defense against microbes [7]. The existence of endocytosis was definitively demonstrated in the 1950s by electron microscopic studies of George Palade [8]. Yet, for about two decades, endocytosis was viewed as a nonspecific process that transports fluid and solutes into cells. In the 1970s, the fundamental observation that nutrients and hormones specifically bind to cells led to the assumption that cells have specific receptors on their surfaces for the uptake of extracellular molecules. This finding generated a milestone paper in endocytic research, namely the discovery of the Low-Density Lipoprotein (LDL) receptor and the description of (LDL) receptor-mediated endocytosis by Anderson, Goldstein, and Brown [9]. This pioneering work put endocytosis at the center of cell biology and disease mechanisms. The motivation behind the identification of this mechanism was the study of the familial hypercholesterolemia (FH), a human genetic disease. Due to the diversity of functions of endocytic mechanisms and their implication in human diseases, endocytosis has become and still is a very active research area. A key point in understanding endocytic mechanisms is to develop probes, methods, and equipment to track them in live cells and tissues. The advantage of endocytosis occurring at the cell surface has allowed researchers to take advantage of optical tools to identify and monitor protein internalization over time [10]. Moreover, the use of monolayered primary cultures and cell lines has permitted to easily access and manipulate receptors on the cell surface. Finally, modern labelling tools and advanced microscopy technology has recently guaranteed a more precise visualization of recycled cargoes or intracellular organelles [11]. In this review, we will first introduce the major endocytic pathways and their association with disease, in particular neurological disorders and cancer. We will then focus on the current technical strategies used to visualize endocytosis in vitro and in vivo, highlighting strengths and weaknesses. 

## 2. Endocytic Pathways

**Figure 1:**
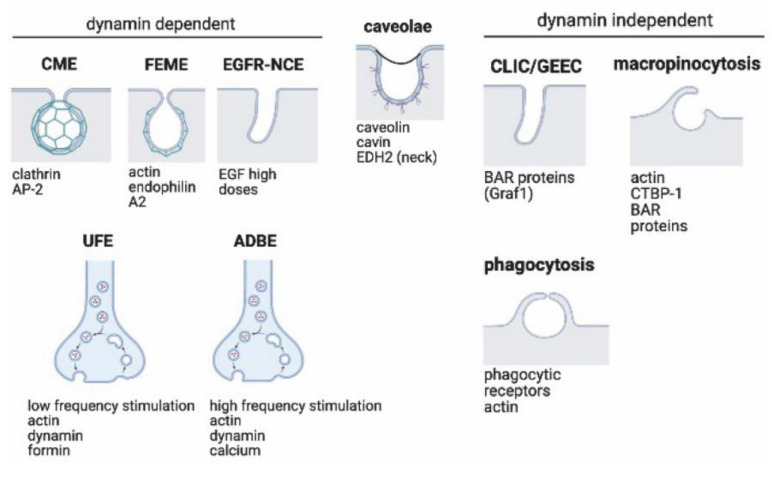
**Overview of primary endocytic pathways in cells.** Endocytosis mechanisms can be additionally classified upon their dependency on dynamin to end the internalization process. Dynamin-dependent processes include CME, FEME, EGFR-NCE, ADBE, and UFE. Clathrin-mediated endocytosis (CME) is driven by the adaptor complex, AP2, that recruits clathrin to cytosolic receptor domains, initiating the formation of a clathrin-coated pit. Fast endophilin-mediated endocytosis (FEME) is triggered by ligand–receptor interactions and regulated by endophilin A2 recruitment and actin polymerization. EGFR-mediated not-conventional endocytosis (EGFR-NCE) is an unconventional clathrin-independent process which occurs when EGF is present in high doses in the extracellular environment. Ultrafast endocytosis (UFE) mediates the recycling of synaptic vesicle components in a clathrin-independent but dynamin-dependent way during very low frequency stimulation, while activity-dependent bulk endocytosis (ADBE) is the dominant mode of synaptic vesicle endocytosis during high-frequency stimulation. Uncertain, instead, is the dependency on dynamin by caveolae. Formation of caveolae is dependent on caveolin and cavin proteins. The EHD2 protein is also important in stabilizing caveolae neck formation. CLIC/GEEC endocytosis is a constitutive clathrin and dynamin independent process, but actin and BAR protein-dependent (e.g., Graf1). Macropinocytosis is controlled by actin dynamics and different BAR domain proteins. Macropinosome fission from the surface is regulated by C-terminal-binding protein 1 (CTBP1). Phagocytosis occurs after a binding event at the cell surface triggers actin polymerization and a vesicle form tightly around the bound material. Created with BioRender.com (accessed on 15 March 2022).

Several endocytic routes allow nutrients, liquids, compounds, receptors, and pathogens to enter cells [12]. Each pathway (except for caveolae) presents characteristic cargoes, including cytosolic markers, endocytic machineries, ligands, and receptors, which use them to enter the cell (Figure 1). Based on their dependence or independence on GTPase dynamin for vesicle fission, endocytic pathways might be distinguished as dynamin-dependent and dynamin-independent. Among dynamin-dependent pathways, we will describe clathrin-mediated endocytosis (CME), fast endophilin-mediated endocytosis (FEME), EGFR non-clathrin endocytosis (EGFR-NCE), neuronal-specific activity-dependent bulk endocytosis (ADBE), and (10) ultrafast endocytosis (UFE). Among dynamin-independent pathways, we will describe clathrin-independent carrier (CLIC) endocytosis/glycosylphosphatidylinositol-anchored protein enriched early endocytic compartment (GEEC) endocytosis, IL2Rβ uptake, micropinocytosis, and phagocytosis. As caveolae/raft dependent endocytosis is still controversial and does not require dynamin, it will be reported as a separate mechanism. 

### 2.1. Dynamin-Dependent Pathways

#### 2.1.1. Clathrin-Dependent Endocytosis

**Figure 2:**
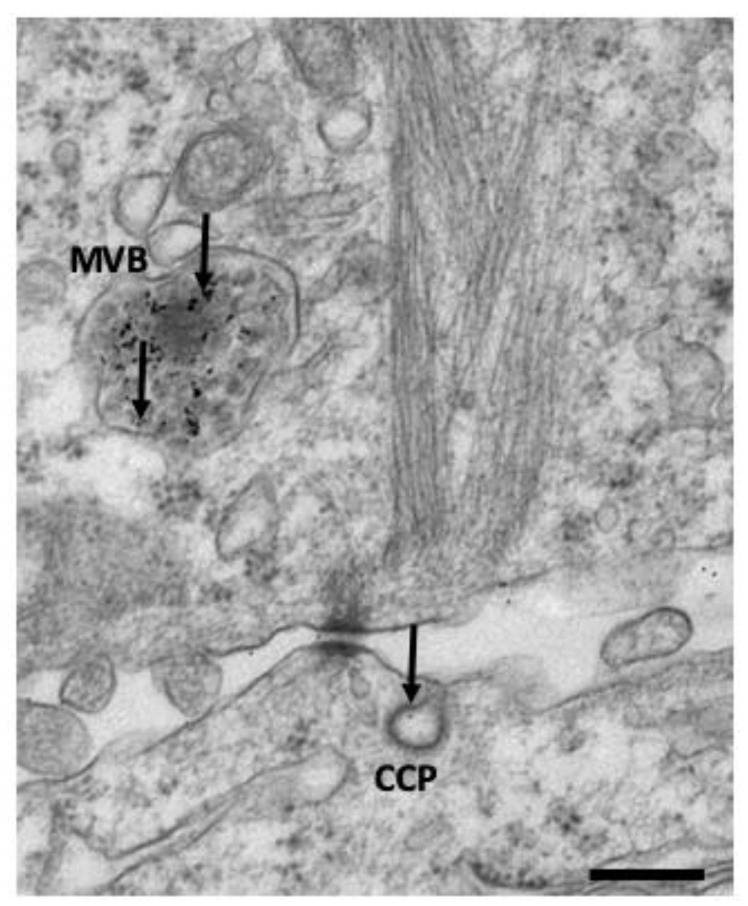
Clathrin-coated pit (CCP) at the plasma membrane and multivescicular bodies (MVB) filled with the endocytic probe BSA conjugated with 5 nm colloidal gold particles (black arrows) of epoxy resin flat embedded ERBB2 + BT474 breast cancer cells. This image was taken by K.C. and represents the lab’s unpublished material.

Clathrin-mediated endocytosis (CME) represents the most characterized internalization pathway [12]. It occurs in all mammalian cells and is the principal route for cells to obtain nutrients; for example, facilitating the uptake of iron (via transferrin) and cholesterol (via low-density lipoproteins) [9] (Figure 2). 

Although historically considered a receptor-induced process, it is now known that clathrin coats can spontaneously assemble at the PM and that cargo-to-clathrin interactions are important for the stabilization of the process. Clathrin-coated pits occupy 0.5–2% of the cell surface and provide the membrane-deforming scaffold, which is fundamental in shaping the coated pit at the plasma membrane [13]. At the PM, clathrin assembles into a trimer of heterodimers, each unit consisting of one heavy and one light chain forming a triskelion [14]. Triskelia assembly lead to the formation of a lattice-like structure around the vesicles and such assembly is coordinated by several cargo-binding adaptor complexes; the most known of these is represented by the adaptor protein complex 2 (AP2), which is part of a wider family of hetero-tetrameric adaptor complexes (AP1-5). AP2 binds both to clathrin and protein cargoes via a peptide motif in their cytoplasmic domains. Alternatively, clathrin adaptors can recruit client cargoes more selectively [15]. These events are also strongly regulated by local actin and phosphoinositides on the plasma membrane. For instance, protein receptors clustering and phosphorylation recruits adaptin proteins at the plasma membrane, which initiates a cascade of low-affinity protein–protein and protein–lipid interactions (particularly with phosphatidylinositol 4,5-bisphosphate, PtdIns(4,5)P2), leading to the formation of a clathrin-coated pit (Smith et al., 2017). This is a highly dynamic and cooperative system in which a multitude of interactions form a pit within 30–120 s of ligand binding [16]. Clathrin-coated pits at the cell surface are highly diverse and with respect to the usage of adaptor and associated proteins [17]. This creates distinct microenvironments for the regulated entry of specific combinations of cargoes [13,18]. Moreover, ligand concentration also affects the mode of this endocytic route. For example, EGF is generally internalized by a clathrin-dependent endocytic route, but at higher concentrations, it enter cells through clathrin-independent routes [18]. Once formed, the pit rapidly invaginates to form a clathrin-coated vesicle, which pinches off the plasma membrane through the activity of dynamin, a large mechanical GTPase. PtdIns(4,5)P2 phosphatases, notably synaptojanin, complete the vesicle cycle by uncoating the vesicles [19]. Several viral pathogens such as the recent SARS-CoV-2 coronavirus and well-characterized families of virus (e.g., alpha-, rhabdo-, flavi-, picorna-, pox-, and adenoviruses, among others) enter cells by clathrin-mediated endocytosis, targeting receptors, or machineries internalized by the clathrin-dependent pathway [20,21]. For some pathogens, this route is obligatory, and for others, CME is one of the available escaping routes. Bacteria and large particles up to 1 μm in diameter have also been shown to co-opt clathrin and form actin-rich pedestals to facilitate their uptake [21]. Although longer than the diameter of the typical clathrin-coated vesicle, these pathogens can be internalized by CME through the actin elongation of the clathrin-coated pit [22]. The requirement for actin recruitment, although, can slow the endocytic process, leading to altered internalization kinetics, compared to conventional CME [23]. 

#### 2.1.2. Fast Endophilin-Mediated Endocytosis (FEME) 

FEME recently emerged as a novel fast endocytic pathway of specific membrane receptors, which are important in cell migration and growth factor signaling [24]. Cargoes that follow FEME include β1-adrenergic, dopaminergic, and acetylcholine receptors; the IL-2 receptor and growth factor receptors (EGFR, HGFR); and toxins such as CTxB and STxB. However, so far, only the β1-adrenergic receptor relies on FEME. The FEME pathway is clathrin-independent, but dynamin dependent. Unlike CME, FEME is not constitutive but is rapidly triggered by binding of receptors by their ligands. Similar to CME, FEME requires a pre-enrichment of its main component, endophilin-A2, into discrete clusters on the plasma membrane prior to receptor activation. Such protein interactions recruit the PtdIns(3,4)P2-binding protein lamellipodin, which stabilize endophilin at the edge of the migrating cells. Formation of FEME carriers is extremely rapid (<10 s). Upon receptor activation, indeed, the direct interaction between the SH3 domain of endophilin and cargoes or the indirect association through intermediate proteins such as CIN85 and Cbl activate a cascade of intracellular signaling, culminating in the formation of a 60–80 nm tubular invagination [23]. In the absence of receptor activation, endophilin spots are rapidly unclustered from the plasma membrane. Recently, Cdk5 and GSK3β were identified as key negative regulators of FEME, allowing the cells rapid uptake by the pathway only when their activity is low. Indeed, Cdk5 and GSK3β antagonize the binding of Endophilin to Dynamin-1 and to CRMP4 for local regulation of FEME [25].

#### 2.1.3. EGFR Non-Clathrin Endocytosis

Although traditionally internalized by CME, EGFR receptors can be internalized by alternative endocytic routes in an activity-dependent manner. High concentrations of EGF (>2 ng/mL) can trigger the EGFR receptor to enter cancer cells using an unconventional clathrin-independent pathway called EGFR-NCE identified by light and electron microscopy [26,27]. At even higher EGF concentrations (>50 ng/mL), both FEME and micropinocytosis mediates rapid EGFR internalization from the cell surface required to protect cells from excessive ERK and AKT signaling [28]. EGFR-NCE occurs via the mono-ubiquitination of EGFR and the release of IP3-mediated Ca^2+^ release stored in the endoplasmic reticulum (ER), which triggers the carrier formation. Moreover, the co-internalization of at least one CD147 receptor is required to internalize EGFR via EGFR-NCE [28,29].

#### 2.1.4. Ultrafast Endocytosis (UFE) 

Ultrafast endocytosis (UFE) is a rapid endocytic route for synaptic vesicles, which have been recently observed both in primary neurons and acute brain slices [30]. UFE occurs within 100 ms from the end of an action potential and generates several 80 nm small and elongated cisternae at the plasma membrane, in proximity of the fusion site [31]. Once internalized, cisternae fuse with synaptic endosomes from which synaptic vesicle are reformed. This process is clathrin-mediated [32]. Although most of the mechanisms that govern UFE are still not fully understood, it is known that (1) UFE is triggered by Ca^2+^, (2) is sensitive to membrane tension and that (3) endophilin, synaptojanin, and dynamin, as well as actin, are important for cisternae membrane curvature [19]. Moreover, local protein organization, as well as lipid composition favoring membrane fluidity, are likely to support UFE [33]. Putative UFE cargoes are represented by synaptic vesicle proteins, which are critical for vesicle function and need thus to be recycled rapidly, such as SNAREs or glutamate transporters. However, how such cargoes are sorted back to the cisternae is still unknown. 

#### 2.1.5. Activity-Dependent Bulk Endocytosis (ADBE)

In neurons, CME and ultrafast endocytosis (UFE) recycle synaptic vesicles in response to low to moderate frequency of action potentials. During sustained neuronal activity, a different endocytic process called activity dependent bulk endocytosis (ADBE) takes over. ADBE resembles micropinocytosis but is triggered by elevated local calcium within synaptic terminals and a high amount of exocytosed membrane [34]. This process is dynamically regulated by two kinases, Cdk5 and Glycogen Synthase Kinase 3b (GSK3b), which inhibit ADBE modulators in resting neurons. During high neuronal activity, the local increase in Ca^2+^ activates the phosphatase calcineurin, which in turn activates ADBE modulators [35]. Once triggered, ADBE generates large actin-driven membrane invaginations, namely bulk endosomes, at the plasma membrane, where newly-released synaptic vesicle cargoes are located. VAMP4 is the main ADBE cargo, but many other synaptic vesicle proteins can be nonspecifically retrieved on the large (up to 500 nm) bulk endosomes [36].

### 2.2. Dynamin-Independent Pathways

A growing number of endocytic pathways do not rely on coat proteins or on a pinching system. Most of these pathways do not need coat assembly even during the endocytic intermediate steps. This is guaranteed by the involvement of lipid or protein components, which are sufficient to initiate membrane deformation without defined coat proteins. Shiga toxin entry, for instance, is one such example where binding of toxin to the ganglioside, Gb3, induces invaginations in cells as well as model membranes [37]. Similarly, endocytosis of many lipid-anchored proteins such as glycosylphosphatidylinositol-anchored proteins (GPI-APs) also does not appear to require any of the well-characterized coat proteins [38]. However, the GPI-anchored prion protein (PrP) might be internalized through both CIE and CME endocytosis, depending on the expression of the LRP-1 receptor, which drives PrP to CME [39,40]. The two main features that distinguish these pathways are the dependency on dynamin and the main protein mediators involved. In the next paragraphs, we will briefly discuss them.

#### 2.2.1. Clathrin-Independent/Dynamin-Independent Endocytosis, CLICs/GEEC

Clathrin-independent carrier (CLIC) endocytosis/glycosylphosphatidylinositol-anchored protein enriched early endocytic compartment (GEEC) endocytosis is a cholesterol/GRAF1-dependent, but clathrin and dynamin-independent endocytic route [41,42]. Similar to FEME, CLIC/GEEC endocytosis occurs at the edge of migrating cells and involves tubular carriers. However, unlike FEME, CLIC/GEEC, endocytosis is a constitutive pathway that mediates the internalization, among others, of hyaluronic acid receptor (CD44) and glycosylphosphatidylinositol-anchored proteins, the adeno-associated virus 2 (AAV2), as well as fluids and membrane [42]. Moreover, CLICs do not pre-form on the plasma membrane before receptor activation. After pathway activation, CLICs tubules mature into GEECs and such process is modulated by ARF1/GBF1, the actin regulatory complex Arp2/3, and the small GTPase Cdc42. For their maturation, CLICs also require the binding of two specific BAR domain proteins to the membrane, IRSp53 and GRAF1, respectively [43]. In addition to the intracellular machinery, extracellular lectins called galectins also contribute to cluster CLIC cargoes on the plasma membrane prior to their invagination [44]. The CLICs pathway is highly sensitive to changes in membrane tension and can, in turn, regulate plasma membrane tension homoeostasis as well. Such regulation is coordinated by the mechano-transducer protein vinculin [45].

#### 2.2.2. IL2Rβ Uptake 

IL2Rβ receptor is generally internalized by FEME in T cells [46]. However, it can also internalize using a distinct, unconventional route, which involves the WAVE 1 complex [47]. Mechanistically, the recruitment of WAVE1 to the cytosolic tail of IL-2Rβ leads to IL-2Rβ clustering and N-WASP activation. N-WASP activation induces local Arp2/3-mediated actin protrusions, which generate macropinocytic-like endocytic pits. This process is further supported by an intracellular signaling involving PI3K, Rac1, and PAK-1 activation [47]. Although similar to the macropinocytosis process, IL-2Rβ endocytosis generates smaller (<0.5 µm) and confined spherical carriers.

#### 2.2.3. Macropinocytosis and Phagocytosis 

Macropinocytosis and phagocytosis are endocytic processes that involve the internalization of large volume fractions of liquids or large-sized particles. Even though they differ in their nature of induction and mechanisms, these processes share multiple mechanistic similarities, including slow kinetics, major membrane remodeling, and cytoskeleton support [48]. Macropinocytosis is a unique process which rapidly allows the intake of large amounts of fluids in different cell types, including immune cells, epithelial, fibroblasts, neurons, microglia, and cancer cells [49]. Although constitutively active in quiescent circulating cells, macropinocytosis is downregulated in mature immune cells [48]. Macropinosomes can vary in size (0.2 to 10 μm in diameter) and can be modulated by both pathogens and chemical compounds. As macropinocytosis is not identical in different cell types, the most common feature is the strict dependency on actin-polymerization machinery and on both Rac1 and PAK1 recruitment [49]. Despite its importance to physiology, the molecular mechanisms underlying macropinocytosis remain only partly understood. For example, how and which molecules contribute in macropinosome scission from the plasma membrane, instead, is still unknown. This is largely due to the difficulty in studying macropinosomes owing to the lack of unique molecules present in these structures.

Phagocytosis is a universal pathway that involves the uptake of large particles (>0.5 µm), including nutrients and pathogens such as bacteria. Macrophages, neutrophils, monocytes, dendritic cells, and osteoclasts are called professional phagocytes, as they perform phagocytosis with high efficiency [50]. This process requires triggered cell surface membrane deformations that usually encircle the particle, which result in phagosomes formation [51]. Two well-described types of phagocytic processes exist: (i) FcR-mediated engulfment of immunoglobulin G-opsonized particles and (ii) complement receptor CR3-mediated ingestion of C3bi-coated particles [50,51]. FcR-mediated phagocytosis, also known as ‘zipper-like’, is mediated by the binding of FcR receptors to the ligands and the activation of a local signaling response which activates actin rearrangement, membrane extension around the ligand, and finally formation of a protruding cup with a zipper-lock arrangement around the pathogen. CR3-type or ‘trigger-like’ phagocytosis, instead, is generally activated by extracellular chemical compounds or particles, which are then loosely encased in a large membrane vesicle. In this process, actin is activated to create local patches that control cell membrane depression. Once formed, phagosomes are gradually acidified and cargo-degraded. Both phagocytic process and phagosome degradation can be manipulated by pathogens to either promote their internalization or escape their degradation [52].

### 2.3. Caveolar Endocytosis

**Figure 3:**
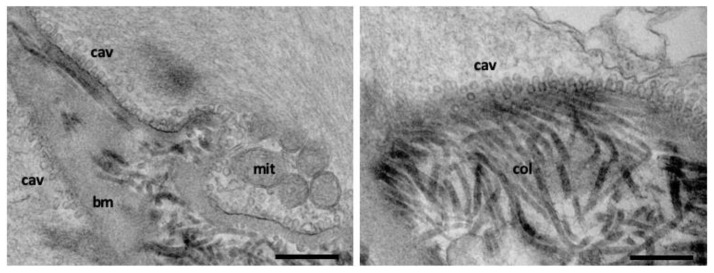
Abundant caveolae at the plasma membrane of vascular smooth muscle cells. Cav: caveolae, mit: mitochondria, bm: basal membrane, col: collagen. Scale bar: 500 nm. These images were taken by K.C. and represents the lab’s unpublished material.

Although controversial, another endocytic process which uses a membrane coat is caveolin. Caveolae are characterized by a unique morphology composed by a bulb-shaped pit of approximately 60–80 nm diameter connected to the plasma membrane by a slightly smaller neck [53]. Structurally, caveolae are formed by assembly of cholesterol binding membrane proteins, termed caveolins, and cytoplasmic protein termed cavins. There are three subtypes of caveolin proteins, two of which are ubiquitarian (cav-1 and cav-2) and one is muscle specific (cav-3). Caveolae formation is cholesterol-dependent and loss in membrane cholesterol leads to disassembly of the caveolar structures. Although highly abundant in some cell types, caveolae are absent in neurons and many blood cells. In cells with abundant caveolae, such as skeletal and smooth muscle, adipocytes, and endothelial cells, recent evidence suggests that caveolae mediate mechano-protection and control of lipid homeostasis, likely acting as a general stress-sensing membrane domain [54,55] (Figure 3). 

Early work focused on caveolar endocytosis for albumin, toxins such as tetanus and cholera toxin, and viruses such as polyoma and simian 40 (SV40) and identified SV40 entry in a specific endocytic structure, termed the caveosome. This compartment had a neutral pH and did not accumulate the lysosomal dye lysotracker [56]. However, more than a decade ago, new elegant evidence provided by the same group demonstrated that caveolae bud, albeit infrequently, from the plasma membrane carrying caveolin and cavins to the classical early endosome, recommending to dismiss the term caveosome [57]. More recently, caveolar endocytosis has been described as a high-efficiency route for cytosolic siRNA delivery of polymeric nanoparticles in macrophages by circumventing lysosomes [58]. Despite these new findings, endocytosis via caveolae remains a controversial issue. It is not clear, for example, whether this endocytic route is constitutive, albeit occurring at a low rate and massively up-regulated upon specific triggers. Insights have been obtained by caveolin knockout mice, which revealed novel roles of caveolin and clarify caveolar cargo specificity [59]. However, the lack of selective specificity for traditional caveolae cargoes observed in knockout mice has raised the hypothesis that caveolin expression level is critical in defining the entry route of certain cargoes. Endocytosis of SV40 virus, for example, has been reported to increase in cells lacking caveolin-1, suggesting an inhibitory role of caveolin-1 in this process [60,61].

## 3. Diseases Caused by Defective Mechanisms of Endocytosis 

Diseases associated with defective endocytosis mechanisms manifest in all tissues. Some of these dysfunctions can be multi-organ and others restricted specifically to one or two. Such diseases often arise from mutations of genes of (i) cargo endocytic proteins, (ii) endocytic machinery components. Depending on the impact of the mutation on the physiology of the tissue, diseases can be developmental or manifest later in aging. In the following paragraphs, we briefly summarize neurological and cancer diseases associated with mutation in endocytic proteins. 

### 3.1. Neurological Diseases

**Figure 4:**
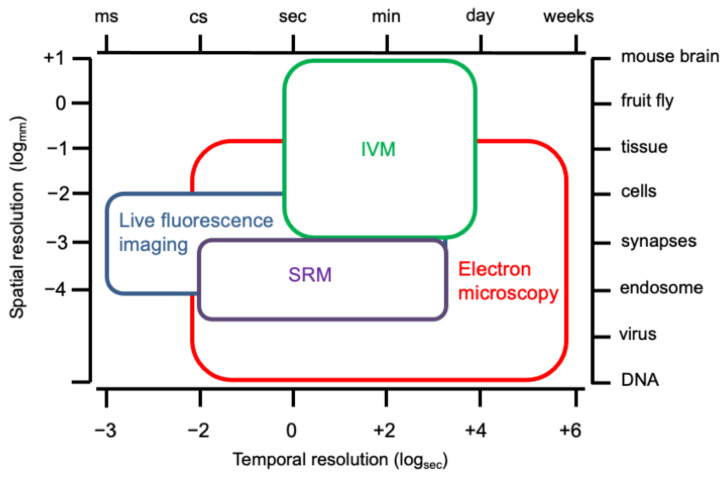
**Spatiotemporal resolution plot for imaging methods to study endocytosis.** Plot recapitulating the estimated spatiotemporal resolution of all the available imaging methods currently used to investigate endocytosis at both in vitro and in vivo dimensions. Methods are shown as colour-coded squares and their size and position reflect their spatiotemporal resolution in the context of investigation of endocytosis. IVM, intravital microscopy; SRM, super-resolution microscopy.

Major neurodegenerative diseases are strongly associated with defects in endocytic pathways, particularly within the endosomal system [62]. Genetic association studies have identified mutations which alter the expression of endocytic machinery genes with Alzheimer disease (AD), Parkinson disease (PD), and amyotrophic lateral sclerosis (ALS). AD-related endocytic genes identified correspond, among others, to the CME-linked phosphatidylinositol-binding clathrin assembly protein (PICALM) [63], CME-linked AP2A1 and AP2A2 [64], CME/FEME-linked bridging integrator 1 (BIN1)/amphiphysin 2 [65], CME-related cortactin-CD2-associated protein (CD2AP) [66], and CME and UFE-related synaptojanin protein [67]. Such mutations can impact AD in multiple ways: for example, by affecting neuron ability in degrading β amyloid or by directly affecting synaptic function [68]. Parkinson’s disease (PD) is also strongly associated with defective endocytosis. Mutation and altered expression of several endocytic proteins, such as cyclin CME related G-associated kinase (GAK) [69], CME-related auxillin [70], and CME/UFE-related synaptojanin [71], have been frequently observed in PD patients. Mutations in endocytic proteins have been also observed in amyotrophic lateral sclerosis (ALS), a neurogenerative disease with results in progressive degeneration of motor neurons. Such mutations include genes such as the RAB5 GEF Alsin (also known as ALS2) [72] and the inositol phosphatase factor induced gene 4 (FIG4), which modulate endocytosis by acting on phosphoinositides synthesis. Moreover, mutations in the CME adaptor SV2A have been widely associated with epilepsy [73]. Lastly, milder mutations in the genes encoding AP-2σ (AP2S1) and AP-2µ (AP2M1) have been identified as cause for familial hypocalciuric hypercalcemia (FHH) type 3 [74] and epileptic encephalopathy [75], respectively. Although many mutations in endocytic genes have been identified, most of the mechanistic causes that lead to disease are still largely unknown. These mutations may affect presynaptic function, channels transport, postsynaptic neurotransmitter receptor availability, and ultimately synaptic protein homeostasis. Further studies will be required to better dissect the impact of disease progression on such mutations.

### 3.2. Cancer 

Endocytosis is intimately linked to cancer as it plays a primary role in transducing intracellular signaling, favoring tumor proliferation and invasiveness, and promoting cell reprogramming [76]. In cells, endocytic organelles act as signaling platforms for different intracellular pathways, including mitogen-activated protein kinase (MAPK), phosphatidylinositol 3 kinase (PI3K), and transforming growth factor b (TGF-b). Such transducing signal is generally activated by the binding of extracellular ligands to tyrosine receptor kinases (RTK) present on the plasma membrane [77]. RTKs are then internalized via different endocytic routes and either degraded or recycled back to the membrane to sustain the intracellular signaling [78,79]. In cancer cells, canonical endocytic pathways that clear RTKs from the plasma membrane or sort RTKs to lysosomes are often compromised [80]. In particular, two famous RTKs families are classically associated with cancer: the ERBB and HGFR families, of which EGFR and cMet are the prototypes, respectively [77]. An escape route to internalization often observed in both RTK families is gene amplification of the receptors. Increased expression of RTKs on plasma membrane prolong their signaling and alter receptors internalization routes, which is often dose-dependent [18]. Head and neck, brain, breast, and other tumors often overexpress ERBB receptors as a result of gene amplification [77]. NSCLC, breast, renal, and ovarian cancer, instead, often present cMet overexpression [81]. Moreover, receptors’ over-expression promotes RTKs’ homo and hetero-dimerization at the plasma membrane, which further reinforce intracellular signaling or receptor recycling. This is the case for the ERBB2 receptor, which is often over-expressed in different tumors, such as breast and gastric tumors [82]. Heterodimerization of EGFR with ERBB2 increases RTK escape from the endocytic routes, as ERBB2 lacks a ligand binding domain and it is therefore often retained to the plasma membrane [83]. Moreover, when ERBB2 is internalized, it rapidly recycles back the plasma membrane, unless it is forced to traffic toward late/lysosomal compartments by anti-neoplastic drugs [84,85]. This, therefore, enhances the recycling of all ERBB receptors associated with it [86]. Another way to escape degradation is by gene deletion of exons that include ligand binding. An EGFR mutant (EGFRvIII) as described often is present in glial tumors [87]. Such mutant can dimerize and activate in the absence of ligand binding. EGFRvIII is inefficiently internalized and degraded but is rather recycled back to the membrane, thus conferring its high tumorigenic potential [88]. Similarly, exon 4 (Ex4) skipping in cMet gene was found in lymph node metastases of head and neck squamous cell carcinomas, non-small cell lung cancer, and associated with metastatic spread and tumorigenesis [89]. Ex4 deletion leads to cMet accumulation on the plasma membrane and persistent signaling [90]. Other mutations in ErbB genes observed in non-small-cell lung cancer include somatic activating mutations in EGFR tyrosine kinase domain, which slow down receptor deactivation [91,92]. Epithelial-mesenchymal transition (EMT) is a critical step in metastatization. Cancer cells loss cell–cell adhesions and apical–basal polarity, and develop a motile phenotype [93]. Such transformation requires a myriad of transcriptional and post-transcriptional events, which include both new protein synthesis and modulation of vesicular trafficking. Endocytosis can modulate polarity changes by refining junctional complexes trafficking. Polarity-maintaining trafficking is regulated by Par and Cdc42 proteins, which are often mutated or down-regulated in tumors [94]. In breast and lung cancers, Arf6 pathway is often up-regulated and represents a risk factor for metastasis when associated with ErbB2 over-expression. This is partially due to the fact that RTKs can regulate trafficking of E-cadherin by themselves [95,96].

## 4. Current Imaging Tools and Techniques to Study Endocytosis in Living Cells and Tissues

To study endocytosis, it is critical to identify and monitor nanometer to micron-sized endocytic structures and their dynamics over time. Over the last 50 years, several optical tools have been developed and further optimized to trace all stages of endocytosis [44]. These include prevalently microscopy approaches and the use of primary cultures or cell lines grown in monolayers, which are easy to access and manipulate. However, the complexity and regulation of endocytic routes increase in complex systems, such as organ cultures and acute slices and even more in live animals, where endocytosis is further regulated by several additional factors (vasculature, extracellular matrix, and hormones), thus requiring specific tools and technologies [10]. In the following paragraph, we discuss different imaging tools and their spatiotemporal resolution used in the study of endocytosis both in vitro and in vivo (Figure 4).

### 4.1. Fluorescence Microscopy (FM)

#### 4.1.1. Internalization Assay by Fluorescence Microscopy

To track a cargo along its preferential internalization pathway, one of the most used tools in cell biology is the internalization assay, which has been developed using either fluorescent probes or labeled antibodies or ligands. The classical internalization assays work as follows: live cells are incubated with the labelled ligand or antibody and, after a defined interval of time, are fixed and the internalized fraction identified by fluorescence microscopy. In such configuration, the fraction of ligand remaining on the cell surface can be specifically removed by wash-out before the fixation or immunolabelled using an antibody after fixation to quantify the net fraction of internalized cargo. These assays are quantitative but can only be used to monitor one moment in the endocytic process at a time. In addition, extreme caution is recommended when the antibody-binding technique is used. It might alter endocytic rates and even endocytic routes due to antibody valency. Alternatively, pulse-chase experiments can be used. However, in order to track receptors over time, it is still required to tag the receptor with a switchable tag, which can simultaneously induce receptor internalization when activated by light or drugs (pulse) and allow to track the receptor in its inactive state (chase) [97,98]. Therefore, to assess the dynamics of the endocytic process over time, live imaging techniques are the preferred ones. 

#### 4.1.2. Fluorescent Probes for Live Fluorescence Imaging 

**Figure 5:**
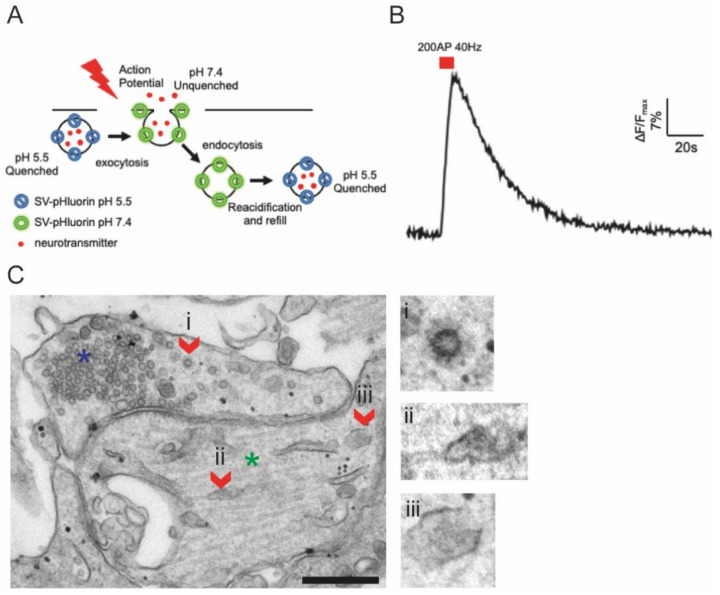
*(***A**). **Schematic representation of an imaging tool based on SEP to study synaptic protein recycling**. Briefly, a vesicular protein tagged with SEP is quenched in the resting condition into acidic SVs (blue). Upon external electrical stimulation that induces action potential firing, SV fuses with PM and increases its fluorescence in a basic environment (green). At the end of the stimulation, SV is internalized back and reacidified (blue). (**B**). Representative synaptic SEP response to an electrical stimulation of 200 APs at 40 Hz (red rectangle) in WT primary cortical neurons expressing Synaptophysin-SEP. An increase in Synaptophysin–SEP fluorescence within seconds from the electrical stimulation corresponds to synaptic vesicle exocytosis. After the stimulation ends, SEP fluorescence decreases, reflecting synaptophysin endocytosis and vesicle acidification. The indicated trace is expressed as ΔF/Fmax, where ΔF is the variation in SEP fluorescence over time respect to the SEP baseline fluorescence (F-F0), and Fmax represents the maximal fluorescence obtained at the end of the stimulation. (**C**). Representative TEM image depicting a synapse from cultured IPSCs cells reprogrammed into neurons at resting. Presynaptic compartment (blue asterisk) can be identified by its enrichment in synaptic vesicles (size 35 nm). Occasionally, clathrin-coated vesicles can be observed (i, red arrow). Post-synaptic compartment (green asterisk), on the contrary, is mainly enriched in elongated or rounded organelles, which resemble bona-fide early endosomes (ii, iii, red arrows). Right, zoomed images of the clathrin-coated vesicle and early endosomes highlighted in the main figure. Scale bar: 400 nm.

A feature shared among all endocytic pathways is that endocytic intermediates (e.g., early endosomes, multivesicular endosomes, late endosomes, and lysosomes) present an increasing acidic environment (from pH 5.5 to 4.5) [99]. Taking advantage of that, several pH-sensitive genetic fluorescent probes for endocytic compartments were designed. A powerful example is the genetic probe pH-sensitive variant of GFP (superecliptic pHluorin, SEP) [100]. Such GFP variants can sense variation in intravescicular pH and decrease their fluorescence when pH undergoes pH 6, thus indirectly reporting endocytosis of membrane cargo proteins when fused in their extracellular domain. This tool has successfully allowed to study synaptic vesicle (SV) endocytosis in synapses and receptor trafficking [101,102]. However, this approach presents several limitations. First of all, this method works efficiently in triggered endocytosis, where differences in fluorescence from resting state are substantially high. However, the abundance of protein cargoes on the plasma membrane and the relative resting fluorescence signal of fused SEP, as well as the constant recycling of cargoes from and to the plasma membrane, produce a background fluorescence that lowers SEPs signal-to-noise ratio and thus limits the visualization of spontaneous endocytic events. In addition, SEP is a genetically encoded tool, and its transfection can induce cell toxicity (Figure 5).

This can be partially overcome by the use of pH-sensitive fluorophores such as CypHer, which behave reversely to SEP and can be conjugated to antibodies [103,104]. CypHer is a red-shifted pH-sensitive cyanine dye whose fluorescence is de-quenched under basic or acidic pH conditions, and quenched under neutral pH conditions. Cypher antibodies against the luminal domain of vesicular proteins can be added directly in the medium and, in response to stimulation, increase in fluorescence upon vesicle endocytosis [105]. The greater advantage of CypHer is then that it does not require cell transfection. However, CypHer has a high bleaching rate, which can compromise endocytosis measurements. Moreover, organelle re-acidification occurs within tens of seconds, therefore limiting the use of both pHluorin and CypHer to slow forms of endocytosis such as CME and some forms of CIE. The most commonly-used pH-sensitive probes used to stain acidic compartments that do not require transfection are fixable Lysosensor probes and DQ-BSA (Dye Quenched-Bovine Serum Albumin), and non-fixable probes like Magic Red (Cathepsin B assay) and the less-specific acridine orange (OA). These tools are designed to fluoresce only in highly acidic lysosomal compartments and are widely used mainly to detect late stages of endocytic trafficking and/or highly acidic compartments (e.g., lysosomal activity in endocytic and autophagic processes). Advantages of these probes include the speed and ease of staining. Limitations of non-fixable probes are that labelling must be performed on living cells/tissue and therefore must be examined immediately; OA also stains nucleic acids [104]. As an alternative to pH-sensitive probes, the dynamics of endocytic associated proteins can be used to indirectly monitor endocytosis using total internal reflection fluorescence (TIRF) microscopy—also known as evanescent wave or evanescent field microscopy. By exciting fluorophores exclusively near the cell surface (within 100 nm), this technique has significantly contributed to gain insight into the components that are necessary for vesicle formation and the dynamics of this process. Using TIRF, several groups have successfully recorded clathrin and dynamin dynamics when fluorescently tagged in cell monolayers. This tool has allowed to understand the dynamics of clathrin uncoating and dynamin recruiting in live cells during CME [105,106]. Moreover, these studies have also demonstrated that some proteins are present throughout the endocytosis process (e.g., clathrin and epsin) [107] and others are critical during specific events [108]. This tool has allowed to understand the dynamics of clathrin uncoating and dynamin recruiting in live cells during CME [105,106]. However, this technique requires transfection and does not precisely measure endocytosis, but rather clustering and dissociation dynamics of its accessory proteins. Moreover, in some cell types, such as neurons, clathrin turnover is much slower than the CME process itself, therefore not perfectly representing this process. This limitation, therefore, makes this tool rather qualitative than quantitative. 

### 4.2. Super-Resolution Microscopy (SRM) Techniques

Although live imaging techniques have provided greater insight into the kinetics of different endocytic pathways, they cannot allow to resolve individual organelles. 

To meet this demand, several super resolution microscopy (SRM) techniques have come to help. Broadly speaking, SRM techniques can be divided into two categories: ensemble SRM techniques, which improve the resolution of overall structures, and single fluorophore SRM techniques, which use individual molecules localization to build an overall structure [109,110]. Stimulated emission depletion (STED), structured illumination microscopy (SIM), and expansion microscopy (ExM) are included in the first category, while single-molecule localization microscopy (SMLM) including direct stochastic optical reconstruction microscopy (dSTORM) and photoactivated localization microscopy (PALM) are single fluorophore techniques.

These techniques have contributed to provide fundamental information on trafficking among different cell types [111,112,113]. For example, SIM microscopy has been useful in revealing that CME and caveolar endocytosis occur at distinctive sites on the plasma membrane [11]. SMLM studies have similarly observed that different receptors on immune cells share communal clustering motifs and that acetylcholine receptors diffuse on neuronal plasma membrane till they are trapped by cytosolic clusters of clathrin and its adapter AP-2. In line with previous investigations, a combination of SMLM, total reflection fluorescence (TIRF), SIM, and STED, has revealed that membrane lipids, e.g., phosphatidylserine and phosphoinositides, also cluster in microdomains in the majority of cell types and that lateral diffusion of receptors is limited by transmembrane proteins [105,106]. Moreover, SRM has also dug out the dynamics of different endocytic pathways. Using SRM correlative microscopy, CME adaptors and actin arrangement during clathrin coat assembly have been mapped, revealing the precise displacement of both actin and accessory proteins in CME [107]. Using fPALM, Mund and Colleagues developed a high-throughput super-resolution microscopy to reconstruct the nanoscale structural organization of 23 endocytic proteins from over 100,000 endocytic sites in yeast [114]. Using this approach, they revealed that nano-scale pre-patterning of actin nucleation present a general design principle for directional force generation in membrane-remodeling processes such as during cell migration and division [114]. SRM has also revealed more insights into caveolin-mediated endocytosis. For instance, SIM and STED have revealed that purinergic receptor P2X7R signaling and Ca^2+^ release from endoplasmic reticulum modulate caveolae rearrangements in osteoblasts [115] and muscles [116], respectively. In some cell types, endocytosis is coupled with exocytosis to dynamically maintain membrane homeostasis. Using SIM microscopy, it was shown that both dynamin and actin are critical players to guarantee an efficient exo–endo coupling [117]. Moreover, at specific cellular compartments, such as synapses, it is still difficult to precisely locate endocytic structures without recurring electron microscopy. The recent advent of expansion microscopy (ExM) has allowed to precisely locate dynamin at the active and pre-active zone of individual synapses in C. Elegans [118]. 

Although SRM has provided impressive improvements in studying endocytic trafficking events, some limitations in spatial, spectral, and temporal resolution need to be resolved. The resolution of super-resolution techniques varies widely—from 100 nm in in-vivo STED, which is still too low to resolve small structures, to the recently developed MINFLUX technique, which has a reported resolution of down to 1 nm [119]. However, antibodies or fused tags (GFP-derived, Halo-tags), conventionally used to visualize endocytic structures, cannot reach that limit spatial resolution due to their size. To overcome this problem, DNA-based point accumulation for imaging in nanoscale topography (DNA-PAINT) technique, has been improved by combining it with smaller molecules [120]. DNA-PAINT uses short dye-labelled DNA oligonucleotides that transiently and specifically interact with their complements, which are attached to the molecule of interest [121,122]. This means that DNA-PAINT can be used for multiplexing, targeting any molecule that can be linked to a docking oligonucleotide strand and to label it via indirect immunolabelling. Therefore, when combined with nanobodies, as well as affimers and novel aptamer probes called SOMAmers, spatial resolution is substantially increased [123,124].

However, more work will be needed to reliably employ these tools in routine SRM. Another drawback of SRM is that most of the techniques can acquire only two colors simultaneously. This is often due to the method used for the image acquisition (beam depletion or fluorophore switching, for example). However, resolving complex endocytic structures require more proteins labelled at the same time. Three and four-channels SRMs are currently being implemented and in the future will be critical in resolving multiple stages of the endocytic process at the same time. Collectively, another limitation of current SRM techniques is that they also present a very low temporal resolution that ranges in seconds and minutes and substantially affects the study of endocytic dynamics in live cells. Novel ongoing developments, such as optical lattice STED (OL-STED) imaging, is demonstrated with a resolution down to 70 nm and will be critical for future temporal monitoring of endocytic events in different cell models [125].

#### In Vivo Imaging and Its Applications to Visualize Membrane Trafficking

Despite significant progress in SRM techniques, their application in living samples are limited due to poor imaging depth. Considerable efforts have been extended to enhancing the imaging depth and developing highly-sensitive fluorescent probes to realize real-time imaging in vivo [126]. For example, Adaptive Optics (AO) has been introduced in many SRM techniques to enhance the imaging depth by eliminating sample-induced distortions [127]. Nowadays, improvements in optical system construction, camera technologies, and labeling methods have made it possible for SRM to visualize physiological activity in living organisms. Although work on primary cell culture and cell lines have extended our knowledge of the underlying molecular mechanisms in endocytosis, it is still unknown whether in vivo cells show the same endocytic mechanisms and how different endocytic pathways contribute to the physiopathology of a specific tissue or organ. Classically investigated by EM, the study of endocytosis in live mammals was recently challenged by recent developments in intra-vital subcellular microscopy (ISMic) methodologies, including the use of light microscopy techniques such as spinning-disc, confocal, and multiphoton microscopy [128]. ISMic combines surgical techniques, organ stabilization tools to reduce motion artifacts, and two or multi-photon microscopy [129]. Two-photon microscopy ensures deep tissue penetration and reduced phototoxicity, which is perfect for the long-term in vivo experiments. ISMic has been extensively used to study tumor progression, but only recently has this tool been used to study endocytosis in vivo. For example, ISMic was successfully used to visualize human squamous cell carcinomas overexpressing EGFR implanted in immunosuppressed mice using carbon nanotubes conjugated with EGF and Qdots [130]. Similarly, the uptake of antibodies against EGFR or EpCAM in colon cancer tumors successfully allow to identify differences in endocytic activity among metastases and primary tumors [130]. Moreover, ISMic permitted the determination that GTPase Rab25 controls tumor cells invasion and dissemination in lymph nodes in vivo by remodeling actin at the plasma membrane and promoting endosomal recycling [131]. However, ISMic has technical spatial and temporal resolution limitations which prevent the visualization of fast forms of endocytosis in vivo. Similar to SRM, novel developments in both optical probes and microscope technology will significantly improve both the spatial and temporal resolution of ISMic techniques [132]. Moreover, ISMic is still limited to a few organs, such as kidney, salivary gland, and few solid tumors. Novel surgical procedures and tools will be needed to access more organs, including the use of microlenses for microendoscopy [133], microstage organ stabilizators [134], and chronic windows for long-term imaging experiments [135].

### 4.3. Electron Microscopy Techniques to Study Endocytosis

From the first characterization of endocytosis by Roth and Porter [136], EM techniques remain one of the most used tools used to investigate the complex membrane structure of endocytic pathways [137]. Here, we briefly describe, together with their strength and weaknesses, the main types of EM tools and techniques that are applied to study endocytic pathways. Classical EM, which involves chemical fixation of cells and tissues, dehydration, embedding in resins, and ultrathin sectioning, was used for decades to study the subcellular morphology of vesicles and organelles [138]. However, in addition to chemical fixation, high-pressure freezing (HPF), followed by freeze substitution, represent an alternative to optimally preserve the ultrastructure of organelles [137]. However, HPF requires expensive equipment and is not easily combined with cytochemical or immunocytochemical methods. In the last decades, EM techniques, based both on chemical and/or HPF fixation methods, allowed to classify and resolve the structure of new types of CIE carriers and caveolae in 2D and 3D [60]. Widely-used and affordable tools to track endocytosis by EM include the labeling of endocytosed structures with soluble or extracellular antibody-conjugated horseradish peroxidase [38,138] or the use of fluid-phase endocytic probes like BSA-colloidal gold conjugated (see Figure 2), used to visualize endocytic intermediates [139]. 

**Figure 6:**
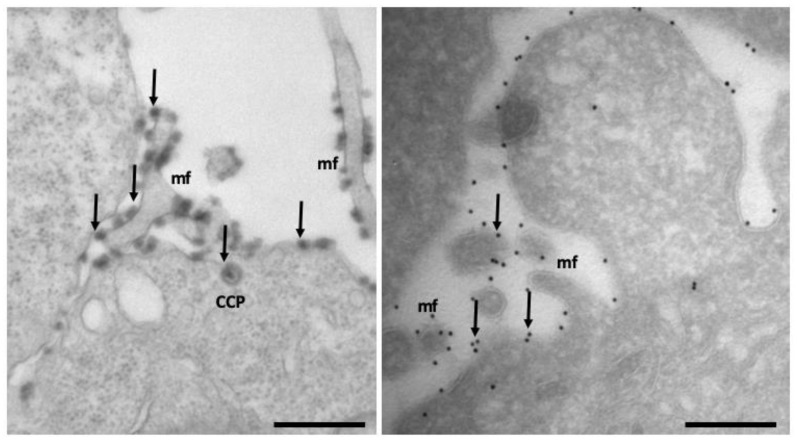
**The ERBB2 receptor visualized by two different immuno–EM techniques**. On the left, ERBB2 is revealed by using HRP-conjugated anti-ERBB2 Trastuzumab antibody in epoxy resin embedded cells; on the right, using an anti-ERBB2 antibody (9G6) revealed by protein A gold 10 nm (arrows) in Tokuyasu cryosections of SKBR3 breast cancer cells. Membrane ruffles (mf, arrows), clathrin-coated pit (CCP, arrow). Scale bar: 500 nm, 200 nm. These images were taken by K.C. and represents the lab’s unpublished material.

Immunoelectron microscopy (IEM) methods allow simultaneous visualization of up to three components of endocytic proteins (both cargo and regulatory molecules) by using antibodies or probes marked with specifically-sized gold particles [139]. A general drawback of immuno-EM techniques is their relatively low sensitivity. When IEM is combined with the Tokuyasu ultrathin cryosectioning technique, most antigens retain their antigenicity. Moreover, because antibodies can to some extent penetrate into the section, labeling efficiency is improved over resin sections. Together with excellent membrane visibility, this makes cryosectioning the IEM method of choice to study the endolysosomal system (Figure 6). The complex and fascinating ultrastructure of the endosomal carriers and network has been better visualized by adding the third dimension by electron tomography (ET). When combined with HPF and freeze substitution in resins, ET is the method of choice to establish contacts and continuities between endosomal compartments and other organelles. Combining ET with protocols that require permeabilization of cells should be interpreted with caution, especially for its impact on structural preservation. The most evident limitation of EM is the impossibility to detect living cells; thus, images represent a specific point in time of the endocytic process, and cannot be used to study endocytic dynamics. In certain cellular models, such as neurons, it is possible to get away with that by coupling EM with advanced genetically-encoded optical tools, such as optogenetics, which allows to obtain snapshots of the endocytic process within hundreds of milliseconds [140]. In particular, the flash and freeze technique is based on a combination of optogenetics that enables to trigger action potential in neurons by expressing a channel rhodopsin, which depolarizes the cells upon light illumination, and results in rapid fixation via the high-pressure method [140]. This method has been successfully used to visualize UFE for the first time, as it allows instantaneous fixation within milliseconds from the end of the light stimulus [141]. 

### 4.4. Correlative Microscopy Techniques to Study Membrane Trafficking

Most light microscopy (LM) studies on the endocytosis use fluorescent probes. These can provide dynamic information from living cells or provide a wide field of view that EM lacks. However, they only visualize fluorescently-labeled components and do not provide information on membrane composition or cellular context. Optimally, one would like to investigate a sample from fluorescence to EM, or even ET, to identify the nature and morphology of the dynamic structures observed by live-cell imaging or peculiar fluorescent patterns seen in fixed cells. As a beautiful example, direct correlation of the dynamics of 211 endocytic proteins with 3D ultrastructural data has been performed by correlative microscopy in budding yeast, precisely defining protein-mediated cell shape changes during endocytosis [142].

Correlative microscopy techniques and novel genetic and/or bimodal probes applied in cell biology have rapidly grown over the last two decades; today, the field has broadened, incorporating preclinical research and diagnostics [143,144]. However, besides the requirement of expensive equipment and expert microscopists, a main limitation of these approaches is achieving a robust quantitative analysis [145,146]. To overcome this limitation, a systematic and quantitative CLEM method performed on Tokuyasu ultrathin cryosections was successfully designed to characterize Rab5, Rab7, EEA1, and APPL1 positive endosomes, classically poorly distinguishable by immuno-EM [147], thus overcoming the throughput problem. New advances in the development of bimodal probes led recently to the development of fluorescent BSA-gold (fBSA-Au), a fluid-phase endocytic tracer. fBSA-Au consists of colloidal gold (Au) particles stabilized with fluorescent bovine serum albumin (BSA). The conjugate is efficiently endocytosed and distributed throughout the 3D endo-lysosomal network of the cells, and has excellent visibility both in fluorescence microscopy (FM) and EM [148]. Performing correlative microscopy in cryo modality, allowing the visualization of structures in their native state, has been also extensively used and resolved, for example, in nanoscale organization of neuronal tunnelling nanotubes (TNTs), structures that mediate intercellular transport of various cargoes [149]. Blooming new technologies, such as volume-scanning EMs (SEMs) based on focused ion-beam scanning electron microscopy coupled with fluorescent imaging, provide the possibility of visualizing large volumes at nanometer resolution in a semi-automated way. An exciting application has enabled the accurate visualization and quantitation of SARS-CoV-2 interaction and endocytosis in cell culture, revealing that SARS-CoV-2 viruses are preferentially located at areas of plasma membrane with positive curvature [150]. 

## 5. Conclusions

Endocytosis is a fundamental process of cell proliferation and function in tissues. Therefore, defects in endocytic machinery often led to tissue-related diseases or multi-organ disorders. Several forms of endocytosis have been identified so far; however, most of the mechanisms involved in such pathways are still unexplored. Although electron microscopy, fluorescence imaging, and super resolution microscopy have significantly contributed in obtaining nanoscale reconstructions of protein, membrane, and lipid organization during endocytic events, novel optical developments and image-analysis algorithms for both in vitro and in vivo investigations are still needed. In particular, algorithms based on deep learning and artificial intelligence [151] will certainly allow for a more precise analysis of fundamental biological questions behind endocytosis, and determine the relative contribution of endocytosis in organ metabolism and diseases, and ultimately promote the development of better therapeutical strategies to cure diseases.

## Data Availability

Not applicable.

## References

[1] Doherty G.J., McMahon H.T. (2009). Mechanisms of Endocytosis. Annu. Rev. Biochem..

[2] Mellman I., Yarden Y. (2013). Endocytosis and Cancer. Cold Spring Harb. Perspect. Biol..

[3] Bonnycastle K., Davenport E.C., Cousin M.A. (2021). Presynaptic Dysfunction in Neurodevelopmental Disorders: Insights from the Synaptic Vesicle Life Cycle. J. Neurochem..

[4] Ballabio A., Gieselmann V. (2009). Lysosomal Disorders: From Storage to Cellular Damage. Biochim. Biophys. Acta Mol. Cell Res..

[5] Yarwood R., Hellicar J., Woodman P.G., Lowe M. (2020). Membrane Trafficking in Health and Disease. DMM Dis. Models Mech..

[6] Kaźmierczak Z., Szostak-Paluch K., Przybyło M., Langner M., Witkiewicz W., Jędruchniewicz N., Dąbrowska K. (2020). Endocytosis in Cellular Uptake of Drug Delivery Vectors: Molecular Aspects in Drug Development. Bioorg. Med. Chem..

[7] Steven J.L. (1892). Metchnikoff on the Comparative Pathology of Inflammation. Glasg. Med. J..

[8] Jamieson J.D. (2008). A Tribute to George E. Palade. J. Clin. Investig..

[9] Anderson R.G.W., Brown M.S., Goldstein J.L. (1977). Role of the Coated Endocytic Vesicle in the Uptake of Receptor-Bound Low Density Lipoprotein in Human Fibroblasts. Cell.

[10] Rosendale M., Perrais D. (2017). Imaging in Focus: Imaging the Dynamics of Endocytosis. Int. J. Biochem. Cell Biol..

[11] Baranov M.V., Olea R.A., van den Bogaart G. (2019). Chasing Uptake: Super-Resolution Microscopy in Endocytosis and Phagocytosis. Trends Cell Biol..

[12] McMahon H.T., Boucrot E. (2011). Molecular Mechanism and Physiological Functions of Clathrin-Mediated Endocytosis. Nat. Rev. Mol. Cell Biol..

[13] Benmerah A., Lamaze C. (2007). Clathrin-Coated Pits: Vive La Différence?. Traffic.

[14] Kirchhausen T., Owen D., Harrison S.C. (2014). Molecular Structure, Function, and Dynamics of Clathrin-Mediated Membrane Traffic. Cold Spring Harb. Perspect. Biol..

[15] Smith S.M., Baker M., Halebian M., Smith C.J. (2017). Weak Molecular Interactions in Clathrin-Mediated Endocytosis. Front. Mol. Biosci..

[16] Ehrlich M., Boll W., van Oijen A., Hariharan R., Chandran K., Nibert M.L., Kirchhausen T. (2004). Endocytosis by Random Initiation and Stabilization of Clathrin-Coated Pits. Cell.

[17] Puthenveedu M.A., von Zastrow M. (2006). Cargo Regulates Clathrin-Coated Pit Dynamics. Cell.

[18] Sigismund S., Woelk T., Puri C., Maspero E., Tacchetti C., Transidico P., di Fiore P.P., Polo S. (2005). Clathrin-Independent Endocytosis of Ubiquitinated Cargos. Proc. Natl. Acad. Sci. USA.

[19] Watanabe S., Mamer L.E., Raychaudhuri S., Luvsanjav D., Eisen J., Trimbuch T., Söhl-Kielczynski B., Fenske P., Milosevic I., Rosenmund C. (2018). Synaptojanin and Endophilin Mediate Neck Formation during Ultrafast Endocytosis. Neuron.

[20] Cossart P., Helenius A. (2014). Endocytosis of Viruses and Bacteria. Cold Spring Harb. Perspect. Biol..

[21] Mercer J., Schelhaas M., Helenius A. (2010). Virus Entry by Endocytosis. Annu. Rev. Biochem..

[22] Veiga E., Cossart P. (2007). Listeria InlB Takes a Different Route to Met. Cell.

[23] Latomanski E.A., Newton H.J. (2019). Taming the Triskelion: Bacterial Manipulation of Clathrin. Microbiol. Mol. Biol. Rev..

[24] Casamento A., Boucrot E. (2020). Molecular Mechanism of Fast Endophilin-Mediated Endocytosis. Biochem. J..

[25] Ferreira A.P.A., Casamento A., Carrillo Roas S., Halff E.F., Panambalana J., Subramaniam S., Schützenhofer K., Chan Wah Hak L., McGourty K., Thalassinos K. (2021). Cdk5 and GSK3β Inhibit Fast Endophilin-Mediated Endocytosis. Nat. Commun..

[26] Sigismund S., Argenzio E., Tosoni D., Cavallaro E., Polo S., di Fiore P.P. (2008). Clathrin-Mediated Internalization Is Essential for Sustained EGFR Signaling but Dispensable for Degradation. Dev. Cell.

[27] Sigismund S., Algisi V., Nappo G., Conte A., Pascolutti R., Cuomo A., Bonaldi T., Argenzio E., Verhoef L.G.G.C., Maspero E. (2013). Threshold-Controlled Ubiquitination of the EGFR Directs Receptor Fate. EMBO J..

[28] Ferreira A.P.A., Boucrot E. (2018). Mechanisms of Carrier Formation during Clathrin-Independent Endocytosis. Trends Cell Biol..

[29] Caldieri G., Barbieri E., Nappo G., Raimondi A., Bonora M., Conte A., Verhoef L.G.G.C., Confalonieri S., Malabarba M.G., Bianchi F. (2017). Reticulon 3-Dependent ER-PM Contact Sites Control EGFR Non-Clathrin Endocytosis Europe PMC Funders Group. Science.

[30] Watanabe S., Boucrot E. (2017). Fast and Ultrafast Endocytosis. Curr. Opin. Cell Biol..

[31] Watanabe S., Rost B.R., Camacho-Pérez M., Davis M.W., Söhl-Kielczynski B., Rosenmund C., Jorgensen E.M. (2013). Ultrafast Endocytosis at Mouse Hippocampal Synapses. Nature.

[32] Watanabe S., Trimbuch T., Camacho-Pérez M., Rost B.R., Brokowski B., Söhl-Kielczynski B., Felies A., Davis M.W., Rosenmund C., Jorgensen E.M. (2014). Clathrin Regenerates Synaptic Vesicles from Endosomes. Nature.

[33] Manni M.M., Tiberti M.L., Pagnotta S., Barelli H., Gautier R., Antonny B. (2018). Acyl Chain Asymmetry and Polyunsaturation of Brain Phospholipids Facilitate Membrane Vesiculation without Leakage. eLife.

[34] Chanaday N.L., Cousin M.A., Milosevic I., Watanabe S., Morgan J.R. (2019). The Synaptic Vesicle Cycle Revisited: New Insights into the Modes and Mechanisms. J. Neurosci..

[35] Cheung G., Cousin M.A. (2013). Synaptic Vesicle Generation from Activity-Dependent Bulk Endosomes Requires Calcium and Calcineurin. J. Neurosci..

[36] Nicholson-Fish J.C., Kokotos A.C., Gillingwater T.H., Smillie K.J., Cousin M.A. (2015). VAMP4 Is an Essential Cargo Molecule for Activity-Dependent Bulk Endocytosis. Neuron.

[37] Römer W., Berland L., Chambon V., Gaus K., Windschiegl B., Tenza D., Aly M.R.E., Fraisier V., Florent J.C., Perrais D. (2007). Shiga Toxin Induces Tubular Membrane Invaginations for Its Uptake into Cells. Nature.

[38] Kirkham M., Fujita A., Chadda R., Nixon S.J., Kurzchalia T.V., Sharma D.K., Pagano R.E., Hancock J.F., Mayor S., Parton R.G. (2005). Ultrastructural Identification of Uncoated Caveolin-Independent Early Endocytic Vehicles. J. Cell Biol..

[39] Parkyn C.J., Vermeulen E.G.M., Mootoosamy R.C., Sunyach C., Jacobsen C., Oxvig C., Moestrup S., Liu Q., Bu G., Jen A. (2008). LRP1 Controls Biosynthetic and Endocytic Trafficking of Neuronal Prion Protein. J. Cell Sci..

[40] Sarnataro D., Caputo A., Casanova P., Puri C., Paladino S., Tivodar S.S., Campana V., Tacchetti C., Zurzolo C. (2009). Lipid Rafts and Clathrin Cooperate in the Internalization of PrPC in Epithelial FRT Cells. PLoS ONE.

[41] Lundmark R., Doherty G.J., Howes M.T., Cortese K., Vallis Y., Parton R.G., McMahon H.T. (2008). The GTPase-Activating Protein GRAF1 Regulates the CLIC/GEEC Endocytic Pathway. Curr. Biol..

[42] Howes M.T., Kirkham M., Riches J., Cortese K., Walser P.J., Simpson F., Hill M.M., Jones A., Lundmark R., Lindsay M.R. (2010). Clathrin-Independent Carriers Form a High Capacity Endocytic Sorting System at the Leading Edge of Migrating Cells. J. Cell Biol..

[43] Sathe M., Muthukrishnan G., Rae J., Disanza A., Thattai M., Scita G., Parton R.G., Mayor S. (2018). Small GTPases and BAR Domain Proteins Regulate Branched Actin Polymerisation for Clathrin and Dynamin-Independent Endocytosis. Nat. Commun..

[44] Rennick J.J., Johnston A.P.R., Parton R.G. (2021). Key Principles and Methods for Studying the Endocytosis of Biological and Nanoparticle Therapeutics. Nat. Nanotechnol..

[45] Thottacherry J.J., Kosmalska A.J., Kumar A., Vishen A.S., Elosegui-Artola A., Pradhan S., Sharma S., Singh P.P., Guadamillas M.C., Chaudhary N. (2018). Mechanochemical Feedback Control of Dynamin Independent Endocytosis Modulates Membrane Tension in Adherent Cells. Nat. Commun..

[46] Lamaze C., Dujeancourt A., Baba T., Lo C.G., Benmerah A., Dautry-Varsat A. (2001). Interleukin 2 Receptors and Detergent-Resistant Membrane Domains Define a Clathrin-Independent Endocytic Pathway. Mol. Cell.

[47] Grassart A., Dujeancourt A., Lazarow P.B., Dautry-Varsat A., Sauvonnet N. (2008). Clathrin-Independent Endocytosis Used by the IL-2 Receptor Is Regulated by Rac1, Pak1 and Pak2. EMBO Rep..

[48] Kerr M.C., Teasdale R.D. (2009). Defining Macropinocytosis. Traffic.

[49] Lin X.P., Mintern J.D., Gleeson P.A. (2020). Macropinocytosis in Different Cell Types: Similarities and Differences. Membranes.

[50] Uribe-Querol E., Rosales C. (2020). Phagocytosis: Our Current Understanding of a Universal Biological Process. Front. Immunol..

[51] Kumari S., Mg S., Mayor S. (2010). Diversity of Endocytic Mechanisms 256 Endocytosis Unplugged: Multiple Ways to Enter the Cell. Cell Res..

[52] Kinchen J.M., Ravichandran K.S.S. (2008). Phagocytic Signaling: You Can Touch, but You Can’t Eat. Curr. Biol..

[53] Parton R.G., Tillu V., McMahon K.A., Collins B.M. (2021). Key Phases in the Formation of Caveolae. Curr. Opin. Cell Biol..

[54] Parton R.G., Kozlov M.M., Ariotti N. (2020). Caveolae and Lipid Sorting: Shaping the Cellular Response to Stress. J. Cell Biol..

[55] Lo H.P., Hall T.E., Parton R.G. (2016). Mechanoprotection by Skeletal Muscle Caveolae. BioArchitecture.

[56] Pelkmans L., Helenius A. (2002). Endocytosis via Caveolae. Traffic.

[57] Hayer A., Stoeber M., Ritz D., Engel S., Meyer H.H., Helenius A. (2010). Caveolin-1 Is Ubiquitinated and Targeted to Intralumenal Vesicles in Endolysosomes for Degradation. J. Cell Biol..

[58] Song Y., Wu Y., Xu L., Jiang T., Tang C., Yin C. (2021). Caveolae-Mediated Endocytosis Drives Robust SiRNA Delivery of Polymeric Nanoparticles to Macrophages. ACS Nano.

[59] Damm E.M., Pelkmans L., Kartenbeck J., Mezzacasa A., Kurzchalia T., Helenius A. (2005). Clathrin- and Caveolin-1-Independent Endocytosis: Entry of Simian Virus 40 into Cells Devoid of Caveolae. J. Cell Biol..

[60] Renard H.F., Boucrot E. (2021). Unconventional Endocytic Mechanisms. Curr. Opin. Cell Biol..

[61] Xing Y., Wen Z., Gao W., Lin Z., Zhong J., Jiu Y. (2020). Multifaceted Functions of Host Cell Caveolae/Caveolin-1 in Virus Infections. Viruses.

[62] Schreij A.M.A., Fon E.A., McPherson P.S. (2016). Endocytic Membrane Trafficking and Neurodegenerative Disease. Cell. Mol. Life Sci..

[63] Jun G., Naj A.C., Beecham G.W., Wang L.S., Buros J., Gallins P.J., Buxbaum J.D., Ertekin-Taner N., Fallin M.D., Friedland R. (2010). Meta-Analysis Confirms CR1, CLU, and PICALM as Alzheimer Disease Risk Loci and Reveals Interactions with APOE Genotypes. Arch. Neurol..

[64] Nelson P.T., Fardo D.W., Katsumata Y. (2021). The MUC6/AP2A2 Locus and Its Relevance to Alzheimer’s Disease: A Review. J. Neuropathol. Exp. Neurol..

[65] Hu X., Pickering E., Liu Y.C., Hall S., Fournier H., Katz E., Dechairo B., John S., van Eerdewegh P., Soares H. (2011). Meta-Analysis for Genome-Wide Association Study Identifies Multiple Variants at the BIN1 Locus Associated with Late-Onset Alzheimer’s Disease. PLoS ONE.

[66] Hollingworth P., Harold D., Sims R., Gerrish A., Lambert J.C., Carrasquillo M.M., Abraham R., Hamshere M.L., Pahwa J.S., Moskvina V. (2011). Common Variants at ABCA7, MS4A6A/MS4A4E, EPHA1, CD33 and CD2AP Are Associated with Alzheimer’s Disease. Nat. Genet..

[67] Choudhry H., Aggarwal M., Pan P.Y. (2021). Mini-Review: Synaptojanin 1 and Its Implications in Membrane Trafficking. Neurosci. Lett..

[68] Nixon R.A. (2017). Amyloid Precursor Protein & Endosomal-Lysosomal Dysfunction in Alzheimer’s Disease: Inseparable Partners in a Multifactorial Disease. FASEB J..

[69] Nagle M.W., Latourelle J.C., Labadorf A., Dumitriu A., Hadzi T.C., Beach T.G., Myers R.H. (2016). The 4p16.3 Parkinson Disease Risk Locus Is Associated with GAK Expression and Genes Involved with the Synaptic Vesicle Membrane. PLoS ONE.

[70] Olgiati S., Quadri M., Fang M., Rood J.P.M.A., Saute J.A., Chien H.F., Bouwkamp C.G., Graafland J., Minneboo M., Breedveld G.J. (2016). DNAJC6 Mutations Associated with Early-Onset Parkinson’s Disease. Ann. Neurol..

[71] Krebs C.E., Karkheiran S., Powell J.C., Cao M., Makarov V., Darvish H., di Paolo G., Walker R.H., Shahidi G.A., Buxbaum J.D. (2013). The Sac1 Domain of SYNJ1 Identified Mutated in a Family with Early-Onset Progressive Parkinsonism with Generalized Seizures. Hum. Mutat..

[72] Hsu F., Spannl S., Ferguson C., Hyman A.A., Parton R.G., Zerial M. (2018). Rab5 and Alsin Regulate Stress-Activated Cytoprotective Signaling on Mitochondria. eLife.

[73] Kononenko N.L., Haucke V. (2020). Neuronal Functions of Clathrin-Associated Endocytic Sorting Adaptors—From Molecules to Disease. Neuroforum.

[74] Nesbit M.A., Hannan F.M., Howles S.A., Reed A.A.C., Cranston T., Thakker C.E., Gregory L., Rimmer A.J., Rust N., Graham U. (2013). Mutations in AP2S1 Cause Familial Hypocalciuric Hypercalcemia Type 3. Nat. Genet..

[75] Helbig I., Lopez-Hernandez T., Shor O., Galer P., Ganesan S., Pendziwiat M., Rademacher A., Ellis C.A., Hümpfer N., Schwarz N. (2019). A Recurrent Missense Variant in AP2M1 Impairs Clathrin-Mediated Endocytosis and Causes Developmental and Epileptic Encephalopathy. Am. J. Hum. Genet..

[76] Khan I., Steeg P.S. (2021). Endocytosis: A Pivotal Pathway for Regulating Metastasis. Br. J. Cancer.

[77] Hynes N.E., MacDonald G. (2009). ErbB Receptors and Signaling Pathways in Cancer. Curr. Opin. Cell Biol..

[78] Parachoniak C.A., Luo Y., Abella J.V., Keen J.H., Park M. (2011). GGA3 Functions as a Switch to Promote Met Receptor Recycling, Essential for Sustained ERK and Cell Migration. Dev. Cell.

[79] Sigismund S., Lanzetti L., Scita G., di Fiore P.P. (2021). Endocytosis in the Context-Dependent Regulation of Individual and Collective Cell Properties. Nat. Rev. Mol. Cell Biol..

[80] Parachoniak C.A., Park M. (2012). Dynamics of Receptor Trafficking in Tumorigenicity. Trends Cell Biol..

[81] Ma P.C., Tretiakova M.S., Mackinnon A.C., Ramnath N., Johnson C., Dietrich S., Seiwert T., Christensen J.G., Jagadeeswaran R., Krausz T. (2008). Expression and Mutational Analysis of MET in Human Solid Cancers. Genes Chromosomes Cancer.

[82] Iqbal N., Iqbal N. (2014). Human Epidermal Growth Factor Receptor 2 (HER2) in Cancers: Overexpression and Therapeutic Implications. Mol. Biol. Int..

[83] Kennedy S.P., Hastings J.F., Han J.Z.R., Croucher D.R. (2016). The Under-Appreciated Promiscuity of the Epidermal Growth Factor Receptor Family. Front. Cell Dev. Biol..

[84] Austin C.D., de Mazière A.M., Pisacane P.I., van Dijk S.M., Eigenbrot C., Sliwkowski M.X., Klumperman J., Scheller R.H. (2004). Endocytosis and Sorting of ErbB2 and the Site of Action of Cancer Therapeutics Trastuzumab and Geldanamycin. Mol. Biol. Cell.

[85] Castagnola P., Bellese G., Birocchi F., Gagliani M.C., Tacchetti C., Cortese K. (2016). Identification of an HSP90 Modulated Multi-Step Process for ERBB2 Degradation in Breast Cancer Cells. Oncotarget.

[86] Worthylake R., Opresko L.K., Wiley H.S. (1999). ErbB-2 Amplification Inhibits down-Regulation and Induces Constitutive Activation of Both ErbB-2 and Epidermal Growth Factor Receptors. J. Biol. Chem..

[87] Jeuken J., Sijben A., Alenda C., Rijntjes J., Dekkers M., Boots-Sprenger S., McLendon R., Wesseling P. (2009). Robust Detection of EGFR Copy Number Changes and EGFR Variant III: Technical Aspects and Relevance for Glioma Diagnostics. Brain Pathol..

[88] Grandal M.V., Zandi R., Pedersen M.W., Willumsen B.M., van Deurs B., Poulsen H.S. (2007). EGFRvIII Escapes Down-Regulation Due to Impaired Internalization and Sorting to Lysosomes. Carcinogenesis.

[89] di Renzo M.F., Olivero M., Martone T., Maffe A., Maggiora P., de Stefani A., Valente G., Giordano S., Cortesina G., Comoglio P.M. (2000). Somatic Mutations of the MET Oncogene Are Selected during Metastatic Spread of Human HNSC Carcinomas. Oncogene.

[90] van der Steen N., Giovannetti E., Pauwels P., Peters G.J., Hong D.S., Cappuzzo F., Hirsch F.R., Rolfo C. (2016). CMET Exon 14 Skipping: From the Structure to the Clinic. J. Thorac. Oncol..

[91] Pao W., Miller V., Zakowski M., Doherty J., Politi K., Sarkaria I., Singh B., Heelan R., Rusch V., Fulton L. (2004). EGF Receptor Gene Mutations Are Common in Lung Cancers from “Never Smokers” and Are Associated with Sensitivity of Tumors to Gefitinib and Erlotinib. Proc. Natl. Acad. Sci. USA.

[92] Sordella R., Bell D.W., Haber D.A., Settleman J. (2004). Gefitinib-Sensitizing EGFR Mutations in Lung Cancer Activate Anti-Apoptotic Pathways. Science.

[93] Thiery J.P., Acloque H., Huang R.Y.J., Nieto M.A. (2009). Epithelial-Mesenchymal Transitions in Development and Disease. Cell.

[94] Vaccari T., Bilder D. (2009). At the Crossroads of Polarity, Proliferation and Apoptosis: The Use of Drosophila to Unravel the Multifaceted Role of Endocytosis in Tumor Suppression. Mol. Oncol..

[95] Morishige M., Hashimoto S., Ogawa E., Toda Y., Kotani H., Hirose M., Wei S., Hashimoto A., Yamada A., Yano H. (2008). GEP100 Links Epidermal Growth Factor Receptor Signalling to Arf6 Activation to Induce Breast Cancer Invasion. Nat. Cell Biol..

[96] Menju T., Hashimoto S., Hashimoto A., Otsuka Y., Handa H., Ogawa E., Toda Y., Wada H., Date H., Sabe H. (2011). Engagement of Overexpressed Her2 with GEP100 Induces Autonomous Invasive Activities and Provides a Biomarker for Metastases of Lung Adenocarcinoma. PLoS ONE.

[97] Weigert R. (2014). Imaging the Dynamics of Endocytosis in Live Mammalian Tissues. Cold Spring Harb. Perspect. Biol..

[98] Zhou L., Evangelinos M., Wernet V., Eckert A.F., Ishitsuka Y., Fischer R., Nienhaus G.U., Takeshita N. (2018). Superresolution and Pulse-Chase Imaging Reveal the Role of Vesicle Transport in Polar Growth of Fungal Cells. Sci. Adv..

[99] Klumperman J., Raposo G. (2014). The Complex Ultrastructure of the Endolysosomal System. Cold Spring Harb. Perspect. Biol..

[100] Martineau M., Somasundaram A., Grimm J.B., Gruber T.D., Choquet D., Taraska J.W., Lavis L.D., Perrais D. (2017). Semisynthetic Fluorescent PH Sensors for Imaging Exocytosis and Endocytosis. Nat. Commun..

[101] Royle S.J., Granseth B., Odermatt B., Derevier A., Lagnado L. (2008). Imaging PHluorin-Based Probes at Hippocampal Synapses. Methods Mol. Biol..

[102] Adie E.J., Kalinka S., Smith L., Francis M.J., Marenghi A., Cooper M.E., Briggs M., Michael N.P., Milligan G., Game S. (2002). A PH-Sensitive Fluor, CypHer^TM^ 5, Used to Monitor Agonist-Induced G Protein-Coupled Receptor Internalization in Live Cells. BioTechniques.

[103] Hua Y., Sinha R., Martineau M., Kahms M., Klingauf J. (2010). A Common Origin of Synaptic Vesicles Undergoing Evoked and Spontaneous Fusion. Nat. Neurosci..

[104] Pierzyńska-Mach A., Janowski P.A., Dobrucki J.W. (2014). Evaluation of Acridine Orange, LysoTracker Red, and Quinacrine as Fluorescent Probes for Long-Term Tracking of Acidic Vesicles. Cytom. Part A.

[105] Asanov A., Zepeda A., Vaca L. (2010). A Novel Form of Total Internal Reflection Fluorescence Microscopy (LG-TIRFM) Reveals Different and Independent Lipid Raft Domains in Living Cells. Biochim. Biophys. Acta Mol. Cell Biol. Lipids.

[106] Wang J., Richards D.A. (2012). Segregation of PIP2 and PIP3 into Distinct Nanoscale Regions within the Plasma Membrane. Biol. Open.

[107] Rappoport J.Z., Kemal S., Benmerah A., Simon S.M. (2006). Dynamics of Clathrin and Adaptor Proteins during Endocytosis. Am. J. Physiol.-Cell Physiol..

[108] He K., Marsland R., Upadhyayula S., Song E., Dang S., Capraro B.R., Wang W., Skillern W., Gaudin R., Ma M. (2017). Dynamics of Phosphoinositide Conversion in Clathrin-Mediated Endocytic Traffic. Nature.

[109] Nozumi M., Nakatsu F., Katoh K., Igarashi M. (2017). Coordinated Movement of Vesicles and Actin Bundles during Nerve Growth Revealed by Superresolution Microscopy. Cell Rep..

[110] Kusumi A., Suzuki K.G.N., Kasai R.S., Ritchie K., Fujiwara T.K. (2011). Hierarchical Mesoscale Domain Organization of the Plasma Membrane. Trends Biochem. Sci..

[111] Freeman S.A., Goyette J., Furuya W., Woods E.C., Bertozzi C.R., Bergmeier W., Hinz B., van der Merwe P.A., Das R., Grinstein S. (2016). Integrins Form an Expanding Diffusional Barrier That Coordinates Phagocytosis. Cell.

[112] Sochacki K.A., Dickey A.M., Strub M.P., Taraska J.W. (2017). Endocytic Proteins Are Partitioned at the Edge of the Clathrin Lattice in Mammalian Cells. Nat. Cell Biol..

[113] Picco A., Mund M., Ries J., Nédélec F., Kaksonen M. (2015). Visualizing the Functional Architecture of the Endocytic Machinery. eLife.

[114] Mund M., van der Beek J.A., Deschamps J., Dmitrieff S., Hoess P., Monster J.L., Picco A., Nédélec F., Kaksonen M., Ries J. (2018). Systematic Nanoscale Analysis of Endocytosis Links Efficient Vesicle Formation to Patterned Actin Nucleation. Cell.

[115] Gangadharan V., Nohe A., Caplan J., Czymmek K., Duncan R.L. (2015). Caveolin-1 Regulates P2X7 Receptor Signaling in Osteoblasts. Am. J. Physiol.-Cell Physiol..

[116] Wong J., Baddeley D., Bushong E.A., Yu Z., Ellisman M.H., Hoshijima M., Soeller C. (2013). Nanoscale Distribution of Ryanodine Receptors and Caveolin-3 in Mouse Ventricular Myocytes: Dilation of T-Tubules near Junctions. Biophys. J..

[117] Kreft M., Jorgačevski J., Stenovec M., Zorec R. (2018). Ångstrom-Size Exocytotic Fusion Pore: Implications for Pituitary Hormone Secretion. Mol. Cell. Endocrinol..

[118] Yu C.C., Barry N.C., Wassie A.T., Sinha A., Bhattacharya A., Asano S., Zhang C., Chen F., Hobert O., Goodman M.B. (2020). Expansion Microscopy of c. Elegans. eLife.

[119] Eilers Y., Ta H., Gwosch K.C., Balzarotti F., Hell S.W. (2018). MINFLUX Monitors Rapid Molecular Jumps with Superior Spatiotemporal Resolution. Proc. Natl. Acad. Sci. USA.

[120] Pleiner T., Bates M., Trakhanov S., Lee C.T., Schliep J.E., Chug H., Böhning M., Stark H., Urlaub H., Görlich D. (2015). Nanobodies: Site-Specific Labeling for Super-Resolution Imaging, Rapid Epitope- Mapping and Native Protein Complex Isolation. eLife.

[121] Schnitzbauer J., Strauss M.T., Schlichthaerle T., Schueder F., Jungmann R. (2017). Super-Resolution Microscopy with DNA-PAINT. Nat. Protoc..

[122] Lelek M., Gyparaki M.T., Beliu G., Schueder F., Griffié J., Manley S., Jungmann R., Sauer M., Lakadamyali M., Zimmer C. (2021). Single-Molecule Localization Microscopy. Nat. Rev. Methods Primers.

[123] Schlichthaerle T., Eklund A.S., Schueder F., Strauss M.T., Tiede C., Curd A., Ries J., Peckham M., Tomlinson D.C., Jungmann R. (2018). Site-Specific Labeling of Affimers for DNA-PAINT Microscopy. Angew. Chem. Int. Ed..

[124] Strauss S., Nickels P.C., Strauss M.T., Sabinina V.J., Carter J.D., Gupta S., Janjic N., Jungmann R. (2019). Modified Aptamers Enable Quantitative Sub-10-Nm Cellular DNA- PAINT Imaging. Nat. Methods.

[125] Yang B., Przybilla F., Mestre M., Trebbia J.-B., Lounis B. (2014). Large Parallelization of STED Nanoscopy Using Optical Lattices. Opt. Express.

[126] Jing Y., Zhang C., Yu B., Lin D., Qu J. (2021). Super-Resolution Microscopy: Shedding New Light on In Vivo Imaging. Front. Chem..

[127] Booth M.J. (2014). Adaptive Optical Microscopy: The Ongoing Quest for a Perfect Image. Light Sci. Appl..

[128] Ebrahim S., Weigert R. (2019). Intravital Microscopy in Mammalian Multicellular Organisms. Curr. Opin. Cell Biol..

[129] Masedunskas A., Milberg O., Porat-Shliom N., Sramkova M., Wigand T., Amornphimoltham P., Weigert R. (2012). Intravital Microscopy: A Practical Guide on Imaging Intracellular Structures in Live Animals. BioArchitecture.

[130] Bhirde A.A., Patel V., Gavard J., Zhang G., Sousa A.A., Masedunskas A., Leapman R.D., Weigert R., Gutkind J.S., Rusling J.F. (2009). Targeted Killing of Cancer Cells in Vivo and in Vitro with EGF-Directed Carbon Nanotube-Based Drug Delivery. ACS Nano.

[131] Amornphimoltham P., Rechache K., Thompson J., Masedunskas A., Leelahavanichkul K., Patel V., Molinolo A., Gutkind J.S., Weigert R. (2013). Rab25 Regulates Invasion and Metastasis in Head and Neck Cancer. Clin. Cancer Res..

[132] Llewellyn M.E., Barretto R.P.J., Delp S.L., Schnitzer M.J. (2008). Minimally Invasive High-Speed Imaging of Sarcomere Contractile Dynamics in Mice and Humans. Nature.

[133] Barretto R.P.J., Messerschmidt B., Schnitzer M.J. (2009). In Vivo Fluorescence Imaging with High-Resolution Microlenses. Nat. Methods.

[134] Cao L., Kobayakawa S., Yoshiki A., Abe K. (2012). High Resolution Intravital Imaging of Subcellular Structures of Mouse Abdominal Organs Using a Microstage Device. PLoS ONE.

[135] Ritsma L., Steller E.J.A., Ellenbroek S.I.J., Kranenburg O., Borel Rinkes I.H.M., van Rheenen J. (2013). Surgical Implantation of an Abdominal Imaging Window for Intravital Microscopy. Nat. Protoc..

[136] ROTH T.F., PORTER K.R. (1964). Yolk Protein Uptake in the Oocyte of the Mosquito *Aedes Aegypti.* L. J. Cell Biol..

[137] Brown E., Mantell J., Carter D., Tilly G., Verkade P. (2009). Studying Intracellular Transport Using High-Pressure Freezing and Correlative Light Electron Microscopy. Semin. Cell Dev. Biol..

[138] Cortese K., Howes M.T., Lundmark R., Tagliatti E., Bagnato P., Petrelli A., Bono M., McMahon H.T., Parton R.G., Tacchetti C. (2013). The HSP90 Inhibitor Geldanamycin Perturbs Endosomal Structure and Drives Recycling ErbB2 and Transferrin to Modified MVBs/Lysosomal Compartments. Mol. Biol. Cell.

[139] Slot J.W., Geuze H.J. (1981). Sizing of Protein A-Colloidal Gold Probes for Immunoelectron Microscopy. J. Cell Biol..

[140] Li S., Raychaudhuri S., Watanabe S. (2017). Flash-and-Freeze: A Novel Technique to Capture Membrane Dynamics with Electron Microscopy. J. Vis. Exp..

[141] Soykan T., Kaempf N., Sakaba T., Vollweiter D., Goerdeler F., Puchkov D., Kononenko N.L., Haucke V. (2017). Synaptic Vesicle Endocytosis Occurs on Multiple Timescales and Is Mediated by Formin-Dependent Actin Assembly. Neuron.

[142] Kukulski W., Schorb M., Kaksonen M., Briggs J.A.G. (2012). Plasma Membrane Reshaping during Endocytosis Is Revealed by Time-Resolved Electron Tomography. Cell.

[143] Parlanti P., Cappello V. (2022). Microscopes, Tools, Probes, and Protocols: A Guide in the Route of Correlative Microscopy for Biomedical Investigation. Micron.

[144] Walter A., Kleywegt G.J., Verkade P. (2021). Correlative Multimodal Imaging: Building a Community. Methods Cell Biol..

[145] Cortese K., Vicidomini G., Gagliani M.C., Boccacci P., Diaspro A., Tacchetti C. (2012). 3D HDO-CLEM: Cellular Compartment Analysis by Correlative Light-Electron Microscopy on Cryosection. Methods in Cell Biology.

[146] Franke C., Repnik U., Segeletz S., Brouilly N., Kalaidzidis Y., Verbavatz J.M., Zerial M. (2019). Correlative Single-Molecule Localization Microscopy and Electron Tomography Reveals Endosome Nanoscale Domains. Traffic.

[147] van der Beek J., de Heus C., Liv N., Klumperman J. (2022). Quantitative Correlative Microscopy Reveals the Ultrastructural Distribution of Endogenous Endosomal Proteins. J. Cell Biol..

[148] Fermie J., de Jager L., Foster H., Veenendaal T., de Heus C., van Dijk S., ten Brink C., Oorschot V.M.J., Yang L., Li W. (2021). Bimodal Endocytic Probe for Three-Dimensional Correlative Light and Electron Microscopy. SSRN Electron. J..

[149] Sartori-Rupp A., Cordero Cervantes D., Pepe A., Gousset K., Delage E., Corroyer-Dulmont S., Schmitt C., Krijnse-Locker J., Zurzolo C. (2019). Correlative Cryo-Electron Microscopy Reveals the Structure of TNTs in Neuronal Cells. Nat. Commun..

[150] Baena V., Conrad R., Friday P., Fitzgerald E., Kim T., Bernbaum J., Berensmann H., Harned A., Nagashima K., Narayan K. (2021). Fib-Sem as a Volume Electron Microscopy Approach to Study Cellular Architectures in SARS-CoV-2 and Other Viral Infections: A Practical Primer for a Virologist. Viruses.

[151] Speiser A., Müller L.R., Hoess P., Matti U., Obara C.J., Legant W.R., Kreshuk A., Macke J.H., Ries J., Turaga S.C. (2021). Deep Learning Enables Fast and Dense Single-Molecule Localization with High Accuracy. Nat. Methods.

